# Case Report: Upadacitinib in the management of refractory urticarial vasculitis

**DOI:** 10.3389/fmed.2025.1669513

**Published:** 2025-08-26

**Authors:** Maan M. Almaghrabi, Nadeen Kalantan, Alhusain Alshareef, Abdulhadi Jfri

**Affiliations:** ^1^College of Medicine, King Abdulaziz University, Jeddah, Saudi Arabia; ^2^Division of Dermatology, Department of Medicine, Ministry of the National Guard-Health Affairs, Jeddah, Saudi Arabia; ^3^Department of Rheumatology, The Ottawa Hospital, Ottawa, ON, Canada; ^4^College of Medicine, King Saud Bin Abdulaziz University for Health Sciences, Jeddah, Saudi Arabia; ^5^King Abdullah International Medical Research Center, Jeddah, Saudi Arabia

**Keywords:** urticarial vasculitis, Upadacitinib, JAK inhibitor, dermatology, rheumatology

## Abstract

Urticarial vasculitis (UV) is a rare autoimmune condition characterized by persistent urticarial lesions with underlying small vessel leukocytoclastic vasculitis. It often presents with systemic symptoms and poses therapeutic challenges, especially in refractory cases. We report the case of a 39-year-old woman who presented with recurrent episodes of widespread, painful wheals lasting more than 24 h, along with arthralgia, myalgia, blurred vision, and fatigue. Her diagnosis was confirmed by skin biopsy, and she failed multiple lines of immunosuppressive and biologic therapies, including corticosteroids, colchicine, omalizumab, dapsone, rituximab, mycophenolate mofetil, and cyclosporine. After initiating treatment with Upadacitinib 30 mg daily orally, in combination with omalizumab and dapsone, she experienced a dramatic clinical improvement within one month, with near-complete resolution of cutaneous lesions and significant relief of systemic symptoms. This case highlights the potential role of JAK inhibitors, particularly Upadacitinib, as a novel therapeutic option in managing refractory urticarial vasculitis. Further studies are needed to evaluate its long-term efficacy and safety in this context.

## 1 Introduction

Urticarial vasculitis (UV) is a rare autoimmune disease. It is characterized by urticarial lesions along with leukocytoclastic vessel wall invasion and perivascular inflammation. It is often accompanied by systemic symptoms such as arthralgia and fever. Compared to chronic urticaria, it involves inflammation of small blood vessels, which leads to more severe and long-lasting skin lesions with a tendency to leave a bruise-like appearance. Diagnosis and management are usually challenging, and a definitive diagnosis with a skin biopsy is often required. UV is a condition that is difficult to treat and typically requires a combination of immunosuppressive and immunomodulatory medications, with different clinical responses depending on the severity of dermatological and systemic involvement (1, 2). Here we present a patient who exhibited a case of difficult-to-treat urticarial vasculitis.

## 2 Case description

We describe a 39-year-old female with recurrent episodes of skin eruptions that consisted of erythematous wheals over the upper limbs, abdomen, chest, and thighs lasting more than 24 h, associated with a burning sensation. She was eventually diagnosed with urticarial vasculitis, proven by biopsy, which showed leukocytoclastic infiltration of the blood vessel wall along with endothelial swelling and rare eosinophils in the papillary dermal blood vessels. She failed multiple lines of systemic treatment, including colchicine, omalizumab, high-dose prednisolone, dapsone, rituximab, mycophenolate mofetil, and cyclosporine. The patient is being followed up by a multidisciplinary team consisting of Dermatology, Rheumatology, and Immunology and unfortunately no certain clinical, environmental, or behavioral condition was identified or associated with her relapses. She reported urticarial rash all over the body, facial swelling, and edema whenever the symptoms were uncontrolled. The patient also complained of arthralgia, myalgia, blurred vision, and fatigue. She denied constitutional symptoms, prolonged morning stiffness of the joints, joint swelling, malar rash, Raynaud’s phenomenon, oral ulcers, dry mouth, dry eyes, red eyes, eye floaters, cough, shortness of breath, abdominal pain, hematuria, frothy urine, recent infections, or recent travel. She reported no history of any chronic illnesses or other autoimmune diseases such as systemic lupus erythematosus or rheumatoid arthritis and no recent history of drug use. She was started on 30 mg Upadacitinib once daily orally, and prednisolone was tapered to 20 mg by dermatology. The patient was also restarted by Immunology on dapsone 100 mg and received a single dose of 450 mg omalizumab. After 1 month, the patient noted drastic improvement in symptoms, with only small lesions on the chest remaining. The patient had a positive antinuclear antibody (ANA) titer, elevated ESR of 46 mm/h (Reference Range: <20 mm/h) and an elevated CRP of 12.6 mg/dl (Reference range: <10 mg/dl), and thrombocytosis: 477 × 10^9/L (Reference Range: 150–450 × 10^9/L). However, she had normal complement levels. Urine analysis was negative for protein and blood. ANCA, hepatitis B surface antigen, hepatitis C serologies, anti-double-stranded DNA, anti-Ro/La, RF, serum protein electrophoresis, and cryoglobulin levels were all within normal limits. Basic metabolic panels and other components of the complete blood count were normal. Chest X-ray and PFT/spirometry were normal. Upon physical examination, a few small urticarial wheals were present on the chest. The patient expressed significant relief and satisfaction after years of ineffective treatments. She noted a substantial improvement in her quality of life, particularly with reduced fatigue and absence of widespread rashes with the absence of any adverse events associated with Upadacitinib. Furthermore, upon follow up the patient has not had any serious adverse events including infections such as herpes simplex virus infection, hematological toxicity, or venous thromboembolism.

**Figure 1:**
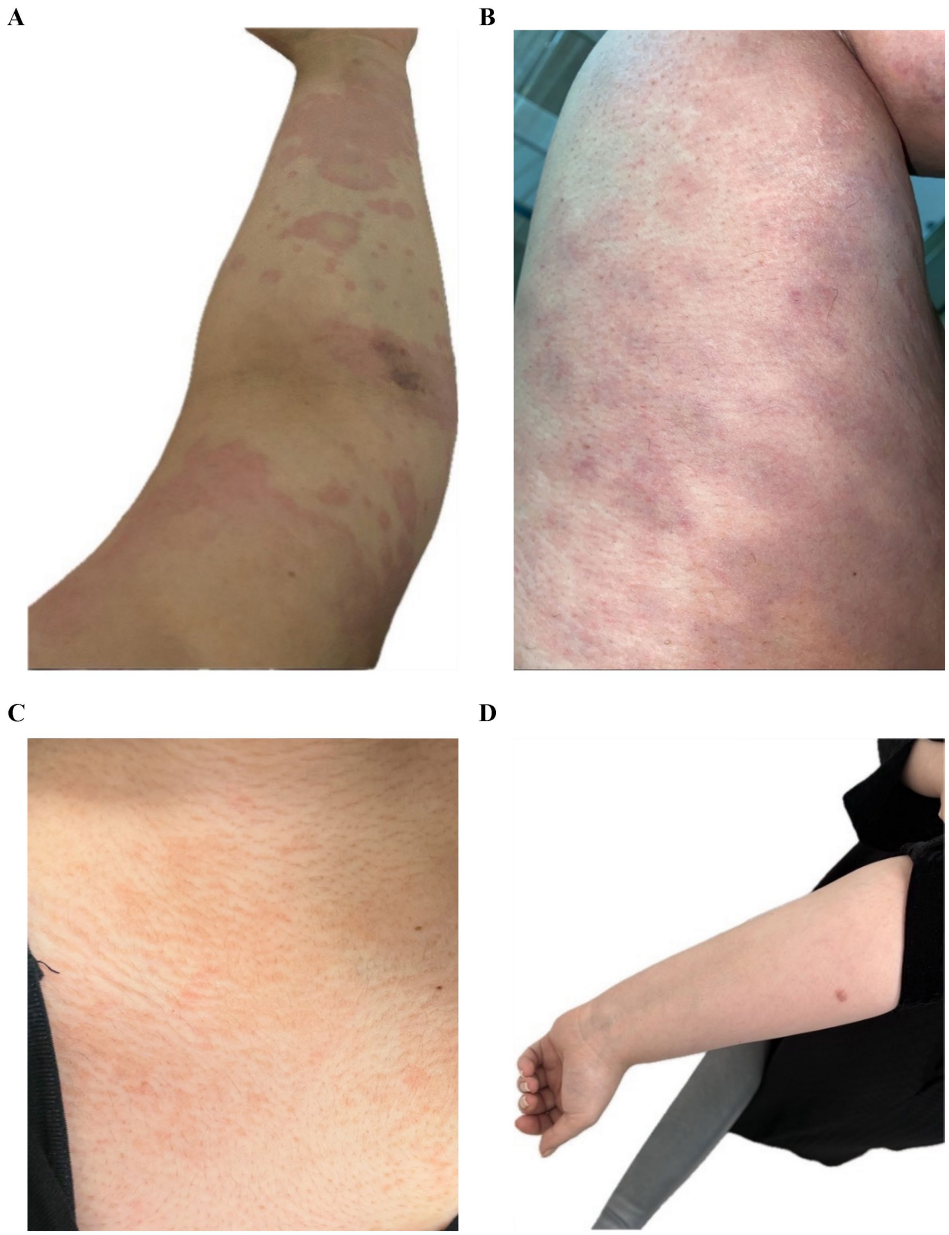
**(A)** Confluent erythematous urticarial wheals on the upper limb. **(B)** Extensive urticarial lesions on the thigh. **(C)** Marked improvement with only a few lesions on the chest. **(D)** Cleared upper limb after treatment.

- Before starting Upadacitinib, the patient had confluent erythematous urticarial wheals all over the body, including the upper limb and thigh (Figures 1A,B).
- After Upadacitinib, only a few lesions remained on the chest and the upper limb cleared (Figures 1C,D).

## 3 Discussion

Urticarial vasculitis is classified into two subtypes based on complement levels: normocomplementemic urticarial vasculitis and hypocomplementemic urticarial vasculitis syndrome (HUVS). HUVS has been associated with a poor prognosis and systemic involvement. UV may overlap with multiple autoimmune disorders, such as systemic lupus erythematosus, rheumatoid arthritis, and Sjögren’s syndrome. Urticarial vasculitis can be differentiated from chronic urticaria as UV lesions typically last 24–48 h, and patients usually present with pain and burning instead of pruritus. Furthermore, the presence of purpura or post-inflammatory hyperpigmentation after the resolution of lesions is consistent with UV (1, 2). Management of severe UV can be challenging because patients may respond poorly to standard regimens. Long-term maintenance with systemic steroids and immunosuppressants is often inappropriate due to their side effects. Upadacitinib is a selective Janus kinase (JAK) inhibitor approved for the treatment of various chronic inflammatory diseases, including dermatological and rheumatological conditions. It inhibits the phosphorylation of downstream proteins and cytokine signaling pathways involved in inflammation, with a greater potency for JAK1 over other isoforms (3, 4). JAK inhibitors have shown efficacy in several dermatological disorders including psoriasis, atopic dermatitis, and vitiligo. There have been case reports discussing the potential benefits of JAK inhibitors in resistant UV and chronic urticaria. One such case involved tofacitinib, which resulted in significant improvement in urticarial lesions by downregulating immune responses involving mast cells (5, 6). Ruxolitinib, an inhibitor of both JAK1 and JAK2, has also demonstrated efficacy in chronic urticaria (7). More recently, two case reports have assessed Upadacitinib as an emerging medication in the treatment of urticarial vasculitis and promising results have been shown. In the case report of Falcinelli et al. a patient was started on 15 mg Upadacitinib with partial improvement in cutaneous symptoms and noted worsening 8 h after the dose. However, when the dose was adjusted to 15 mg twice daily yielding a daily dosage of 30 mg the patient noted complete remission of urticarial lesions and articular symptoms which is a similar result to our case. In contrast the case of Xie et al. noted major improvement upon starting 15 mg Upadacitinib once daily orally and almost all the wheals have disappeared within 3 days of management and no new large wheals appeared and throughout the next 2 weeks a few lesions appeared on the palms and soles, but they faded in a timely manner (8, 9). However, the exact mechanisms by which JAK inhibitors alleviate UV symptoms are not fully understood. Urticaria is believed to be primarily due to mast cell activation and degranulation. The limited effectiveness of anti-IgE therapies like omalizumab suggests that IgE-independent pathways may also play a role. Increasing evidence highlights the importance of proinflammatory cytokine signaling. Thus, inhibition of the JAK/STAT signaling pathway may reduce immune complex formation and vascular deposition, both of which are significant in UV pathogenesis (10, 11).

## 4 Conclusion

In conclusion, Upadacitinib appears to downregulate the immune response associated with urticarial vasculitis. This report has several limitations, as it describes a single case and should be considered preliminary evidence requiring further clinical investigation. Additionally, we did not explore the exact mechanism of action of this drug. Further studies are necessary to evaluate the efficacy of JAK inhibitors, specifically Upadacitinib, in the treatment of urticarial vasculitis.

## Data Availability

The raw data supporting the conclusions of this article will be made available by the authors, without undue reservation.
